# Real-Time Temperature Effects on Dynamic Impact Mechanical Properties of Hybrid Fiber-Reinforced High-Performance Concrete

**DOI:** 10.3390/ma18143241

**Published:** 2025-07-09

**Authors:** Pengcheng Huang, Yan Li, Fei Ding, Xiang Liu, Xiaoxi Bi, Tao Xu

**Affiliations:** 1 College of Civil Engineering, Xuzhou University of Technology, Xuzhou 221018, China; 12241@xzit.edu.cn (P.H.); 11501@xzit.edu.cn (F.D.); 13051865033@163.com (X.B.); xyd18@xzit.edu.cn (T.X.); 2 College of Civil and Harbor Engineering, Jiangsu Ocean University, Lianyungang 222005, China; 2022220425@jou.edu.cn

**Keywords:** high-performance concrete, real-time temperature, SHPB, hybrid fiber-reinforced high-performance concrete, dynamic impact mechanical properties

## Abstract

Metallurgical equipment foundations exposed to prolonged 300–500 °C environments are subject to explosion risks, necessitating materials that are resistant to thermo-shock-coupled loads. This study investigated the real-time dynamic compressive behavior of high-performance concrete (HPC) reinforced with steel fibers (SFs), polypropylene fibers (PPFs), polyvinyl alcohol fibers (PVAFs), and their hybrid systems under thermo-shock coupling using real-time high-temperature (200–500 °C) SHPB tests. The results revealed temperature-dependent dynamic responses: SFs exhibited a V-shaped trend in compressive strength evolution (minimum at 400 °C), while PPFs/PVAFs showed inverted V-shaped trends (peaking at 300 °C). Hybrid systems demonstrated superior performance: SF-PVAF achieved stable dynamic strength at 200–400 °C (dynamic increase factor, DIF ≈ 1.65) due to synergistic toughening via SF bridging and PVAF melt-induced pore energy absorption. Microstructural analysis confirmed that organic fiber pores and SF crack-bridging collaboratively optimized failure modes, reducing brittle fracture. A temperature-adaptive design strategy is proposed: SF-PVAF hybrids are prioritized for temperatures of 200–400 °C, while SF-PPF combinations are recommended for 400–500 °C environments, providing critical guidance for explosion-resistant HPC in extreme thermal–industrial settings.

## 1. Introduction

High-performance concrete (HPC), characterized by its dense microstructure, high strength, and superior durability, demonstrates excellent workability and has become a widely adopted material in fields such as high-rise buildings, bridges, and wind turbine foundations [1,2,3]. With scientific advancements, significant progress has been made in HPC’s self-healing capability [4] and environmental adaptability [5], notably enhancing its applicability in extreme engineering environments. Concurrently, rapid developments in ultra-high-performance concrete (UHPC) and fiber-reinforced concrete (FRC) research are offering new pathways to improve concrete toughness and high-temperature performance [6,7,8].

However, HPC faces significant challenges under high-temperature conditions. Primarily, the evaporation of internal moisture generates substantial high-pressure steam within the matrix, escalating spalling risk [9]. Secondly, dehydration of C-S-H gel, differential expansion from aggregate phase transformations, and micro-crack formation collectively contribute to mechanical degradation [10,11,12]. The existing research confirms that temperature gradients and heating rates critically influence thermo-mechanical responses, with rapid heating exacerbating spalling susceptibility [13,14]. Furthermore, dynamic loading behavior under high temperatures warrants particular attention, as concrete structures in service environments often experience concurrent explosive/impact loads—where dynamic mechanical performance directly governs structural safety [15,16].

The incorporation of fibers or hybrid fiber systems represents an effective approach for enhancing concrete performance under high temperatures [17,18]. Steel, PVA, and PP fibers—along with their binary or ternary hybrid combinations—significantly improve toughness and thermal resistance by modifying crack propagation paths and absorbing fracture energy [19,20,21,22]. Notably, PP fibers melt at elevated temperatures to form micro-channels that effectively release internal vapor pressure, thereby mitigating spalling [22,23,24,25]. The synergistic reinforcement mechanisms of composite fibers have gained increasing attention, with studies confirming that steel–polypropylene combinations substantially enhance residual performance under coupled thermal–impact conditions [26,27,28].

Nevertheless, the current research on high-temperature mechanical properties predominantly relies on conventional post-cooling testing methods [29,30,31], which fail to capture the dynamic response characteristics of concrete under actual thermal environments [32]. While real-time high-temperature impact testing techniques remain in the developmental stages, with most current studies focused on rock specimens [33,34,35,36], research specifically targeting concrete under such conditions is scarce [37]. This gap restricts our comprehensive understanding of fiber-reinforced HPC performance under thermo-impact loading. Furthermore, the degradation mechanisms of fibers at elevated temperatures and their consequent effects on overall concrete performance have yet to be fully elucidated, necessitating complementary investigations integrating microscopic analysis.

To address these research gaps, this study innovatively integrates a real-time thermal module with the SHPB impact testing apparatus to simulate the coupled effects of temperature and dynamic loading in practical engineering scenarios. This study systematically evaluates the impact mechanical properties of HPC with different fiber composite systems under 200–500 °C conditions. Through multifactorial analysis of fiber types, content, and temperature gradients, this work reveals fiber reinforcement mechanisms and failure evolution patterns in concrete under real-time high-temperature environments. The findings provide critical theoretical foundations and technical guidance for concrete design in high-risk thermal–industrial settings, such as metallurgical equipment foundations.

## 2. Materials and Methods

### 2.1. Experimental Materials

**Figure 1:**
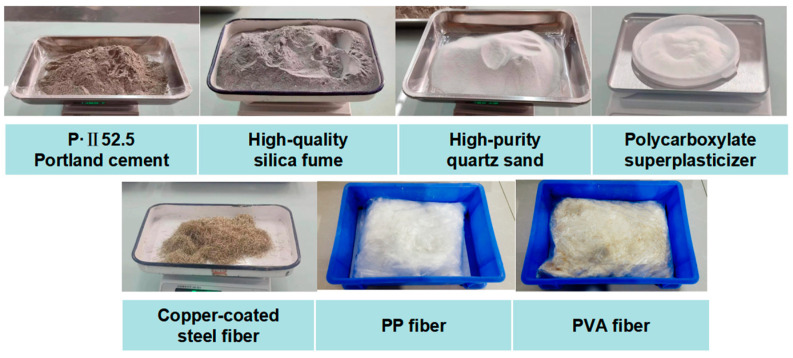
Samples of the test materials.

The used mixture proportions are summarized in Table 1, and the group designations are detailed in Table 2. The raw materials are illustrated in Figure 1.

This study utilized Portland cement (Type P∙Ⅱ52.5, Jiangsu Chengyi Cement Co., Ltd., Xuzhou, China) as the primary binder, supplemented with high-quality silica fume to enhance the matrix density. The fineness of the silica fume was as follows: particle size distribution: >80% of particles finer than 1 μm, median particle size: 0.1–0.3 μm, and specific surface area: 20–28 m^2^/g. High-purity quartz sand (40–70 mesh) served as fine aggregate, and a polycarboxylate superplasticizer was added to control the water-to-binder ratio (fixed at 0.18).

The fibers incorporated in this study comprised three types:

(1) Copper-coated steel fibers (SFs) with a length of 12 mm, a diameter of 0.22 mm, and an elastic modulus of 200 GPa;
(2) Polypropylene fibers (PPFs) with a length of 12 mm, a diameter of 30–40 μm, and an elastic modulus ≥ 3.5 GPa;
(3) Polyvinyl alcohol fibers (PVAFs) with a length of 12 mm, a diameter of 50–60 μm, and an elastic modulus ≥ 25 GPa.

Glass fiber-reinforced specimens were initially included in the experimental design; however, during preliminary heating trials, these specimens exhibited explosive spalling at temperatures below 300 °C. Consequently, data related to glass fiber reinforcement were excluded from the analysis and are not reported herein.

In the base mix design, all three fiber types were added at 2 vol.% individually. Considering the melting susceptibility of PVA and PP fibers above 300–500 °C, hybrid systems were prepared by combining steel fibers (SFs) with PVA or PP fibers in a 1:1 volumetric ratio (1 vol.% SFs + 1 vol.% PVA/PP fibers) while maintaining the total fiber content at 2 vol.%.

### 2.2. Experimental Methods

#### 2.2.1. Specimen Preparation and Static Mechanical Testing

Specimens were cast using a standard mortar mixer (The JJ-5 Cement Mortar Mixer, Wuxi Jianyi Instrument & Machinery Co., Ltd., Wuxi, China) according to test procedures, followed by mechanical vibration compaction (ZS-15 Cement Mortar Vibrating Table, Wuxi Building Materials Instrument Machinery Factory, Wuxi, China). For each fiber combination, nine specimens per group were prepared for static mechanical testing: uniaxial compression tests, Brazilian splitting tests, and three-point bending tests.

The experimental design comprised four temperature levels (200 °C, 300 °C, 400 °C, and 500 °C) and five fiber groups, with three specimens per condition allocated for real-time coupled thermal–impact dynamic testing.

**Figure 2:**
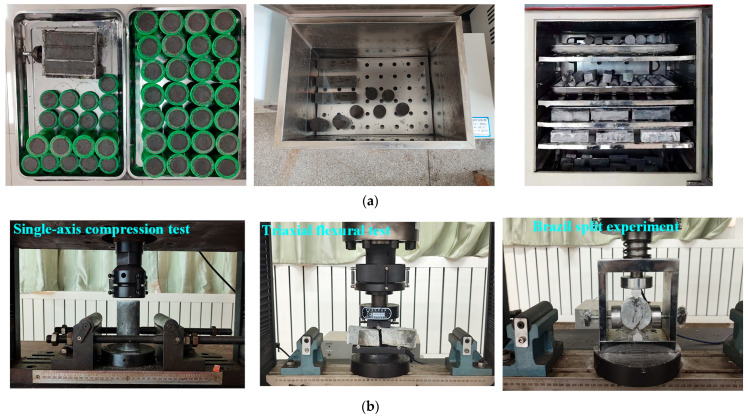

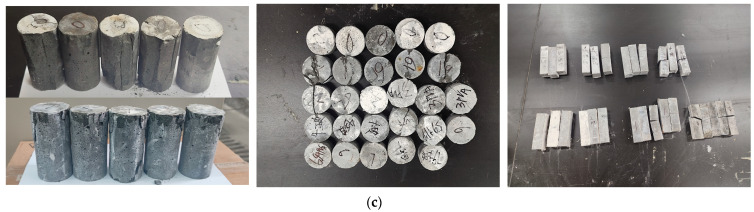
The specimens and the corresponding test processes. (**a**) Schematic diagram of cast specimens undergoing water bath curing and curing chamber maintenance; (**b**) schematic diagram of the static test process; (**c**) partially damaged specimen.

After vibration compaction and surface smoothing, the specimens were cured in a standard chamber (temperature: 20.0 ± 0.5 °C; relative humidity: ≥95%) for 24 h before demolding. Subsequently, they underwent 24 h of hot water bath curing at 70 °C, followed by standard chamber curing until they reached 28-day maturity. The specimen preparation and testing procedures are illustrated in Figure 2. Static mechanical tests followed conventional methods and are not elaborated here.

#### 2.2.2. Real-Time High-Temperature Environment Implementation

**Figure 3:**
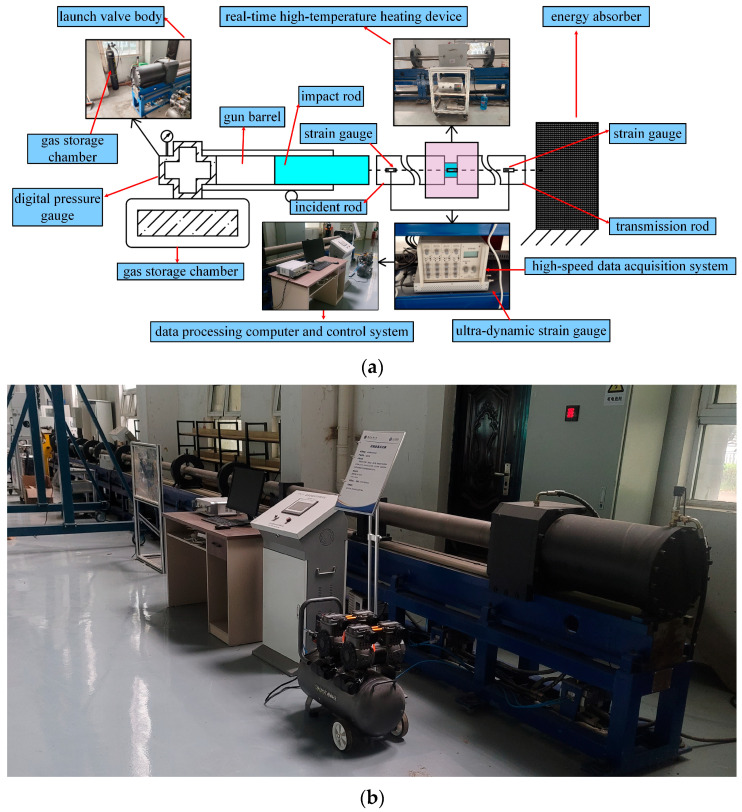

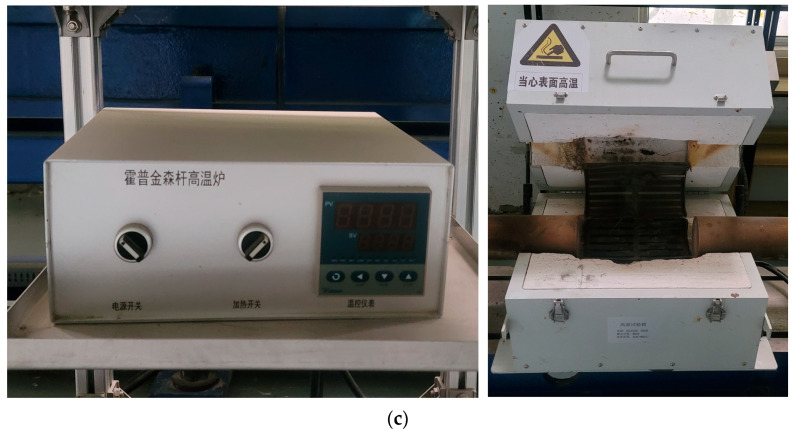
Experimental apparatus. (**a**) Schematic diagram of the SHPB apparatus; (**b**) physical view of the SHPB apparatus; (**c**) temperature control unit and environmental chamber.

To simulate in situ high-temperature conditions, a real-time thermal module was integrated into the SHPB setup. The system comprises a control unit and an environmental chamber, where the control unit regulates chamber temperatures from ambient to 800 °C. Temperature thresholds were set at 200 °C, 300 °C, 400 °C, and 500 °C to align with industrial thermal exposure scenarios, while ensuring comprehensive sampling (Figure 3c).

#### 2.2.3. Impact Testing

The experimental method for impact testing was consistent with conventional SHPB procedures [38,39], except for the integration of the high-temperature coupling module. The specific steps are as follows:

(1) System Preheating and Calibration:

The data acquisition system (Datalab software, ZD-SHPB-V1.0) and strain gauge were activated. After stabilizing the strain gauge readings, channel voltages were zeroed. Acquisition channels, time windows, voltage ranges, and trigger parameters were configured in Datalab software. Continuous acquisition mode was executed to monitor waveform baselines at 0 V. Any detected offset was corrected using the software’s remote zeroing function. Following calibration, the trigger mode was switched to channel triggering.

(2) Specimen Installation and High-Temperature Environment Setup:

Specimens conditioned at target temperatures were extracted from the high-temperature module. Impact surfaces of the incident and transmission bars were uniformly coated with grease to minimize end-friction and wave reflection interference. The specimens were precisely centered between bar ends; thin tungsten wires were used to suspend and secure both specimen ends to prevent slippage, ensuring full contact without visible gaps. The assembled bar–specimen system was transferred to the environmental chamber of the heating module. The chamber was heated to target temperatures (200 °C, 300 °C, 400 °C, or 500 °C) and maintained for ≥30 min to ensure uniform internal temperature distribution.

(3) Impact Loading:

Control software parameters were set as follows: main gas tank pressure (0.2 MPa), bullet reset delay (5–10 s), buffer pressure release time (5 s), and pressure offset (0.01 MPa). The control system was set to automatic mode, and the preparation command was executed. When the “Ready to Launch” status appeared, the single-acquisition mode was initiated in Datalab, displaying “Awaiting Trigger.” The bullet launch was triggered by simultaneously pressing the “Launch Safety Interlock” button on the control screen and the “Launch” switch on the operation panel. The switches were released upon audible confirmation of bullet–bar impact.

(4) Data Acquisition, Processing, and Storage:

Stress wave signals generated by impact were captured by strain gauges bonded to the incident and transmission bars and then transmitted to Datalab via the strain gauge system. The software acquired and displayed raw voltage–time signals of incident, reflected, and transmitted waves in real time. Waveform data files were saved after noise filtering via built-in software. The processed data were subsequently analyzed using dedicated Hopkinson bar analysis software (https://www.zone-de.com/productshow-64-52-1.html (accessed on 6 July 2025)).

#### 2.2.4. Observation Methods

Scanning electron microscopy (SEM) was employed to examine small fragments of specimens subjected to real-time high-temperature impact (Model: VEGA3 TESCAN, TESCAN, s.r.o., Brno, Czech Republic). Microscopic pores, cracks, and fiber distribution were observed to reveal the fiber-induced enhancement and toughening mechanisms. Additionally, crushed specimens were collected to assess the degree of fragmentation, thereby determining the failure modes and energy absorption characteristics of different fiber combinations.

## 3. Results

### 3.1. Static Mechanical Properties at Ambient Temperature

**Figure 4:**
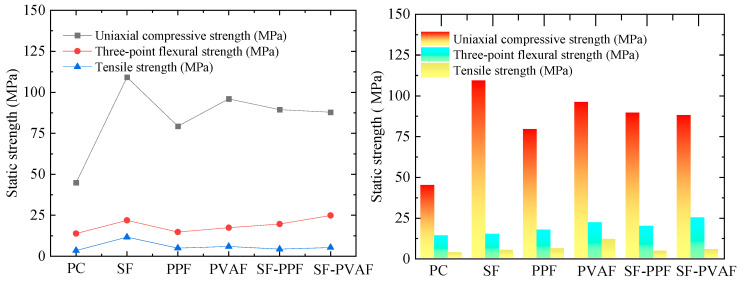
The basic static mechanical properties of different samples at room temperature.

Figure 4 presents the fundamental static mechanical test results of HPC reinforced with different fiber combinations (non-fiber, pure steel fiber (SF), pure polypropylene fiber (PPF), pure polyvinyl alcohol fiber (PVAF), steel–PP hybrid fibers (SF-PPF), and steel–PVA hybrid fibers (SF-PVAF)) under ambient conditions. For easier comparison, the static strength indices of each specimen in Figure 4 are listed in Table 3. As shown in Figure 4, the incorporation of fiber significantly enhanced the static mechanical properties. The following observations were notable:

Uniaxial Compressive and Tensile Strength: The specimens with 2% steel fiber alone exhibited the most pronounced improvement (143.44% and 240.06% increases, respectively).

Flexural Strength: The SF-PVAF hybrid specimens (1:1 volumetric ratio) achieved the highest flexural strength (24.86 MPa, 79.62% improvement), demonstrating optimal multi-scale crack resistance.

### 3.2. Dynamic Mechanical Properties Under Real-Time High Temperature

#### 3.2.1. Electrosignal-to-Stress/Strain Transformation in SHPB Testing

The data processing software used in this test follows the calculation principles based on the two-wave method. The acquired electrical signals (reflected strain *ε_r_*(*t*) and transmitted strain *ε_t_*(*t*)) are converted into stress and strain using the following formulas:

(1) $$\varepsilon_{i}\left(t\right)=\varepsilon_{t}\left(t\right)-\varepsilon_{r}\left(t\right)$$

(2) $$\sigma_{eng}\left(t\right)=\frac{A_{0}}{A_{s}}E\varepsilon_{t}\left(t\right)$$

(3) $$\varepsilon_{eng}\left(t\right)=-\frac{{2C}_{0}}{L_{s}}\int_{0}^{t}\varepsilon_{r}\left(t\right)dt$$

(4) $$\sigma_{true}\left(t\right)=\frac{\sigma_{eng}\left(t\right)}{1-\left|\varepsilon_{eng}\left(t\right)\right|}$$

(5) $$\varepsilon_{true}\left(t\right)=-\ln\left(1-\left|\varepsilon_{eng}\left(t\right)\right|\right)$$

where *ε_r_*(*t*) is the reflected strain, *ε_t_*(*t*) is the transmitted strain, *ε_i_*(*t*) is the incident strain, *ε*_eng_(*t*) is the engineering strain, *ε*_true_(*t*) is the true strain, σ_eng_(*t*) is the engineering stress (Pa), σ_true_(*t*) is the true stress (Pa), *A*_0_ is the cross-sectional area of the pressure bar (m^2^), *A_S_* is the initial cross-sectional area of the specimen (m^2^), *E* is the elastic modulus of the pressure bar (Pa), *C*_0_ is the elastic wave velocity in the pressure bar (m/s), and *L_s_* is the initial length of the specimen (m).

#### 3.2.2. Dynamic Compressive Strength

**Figure 5:**
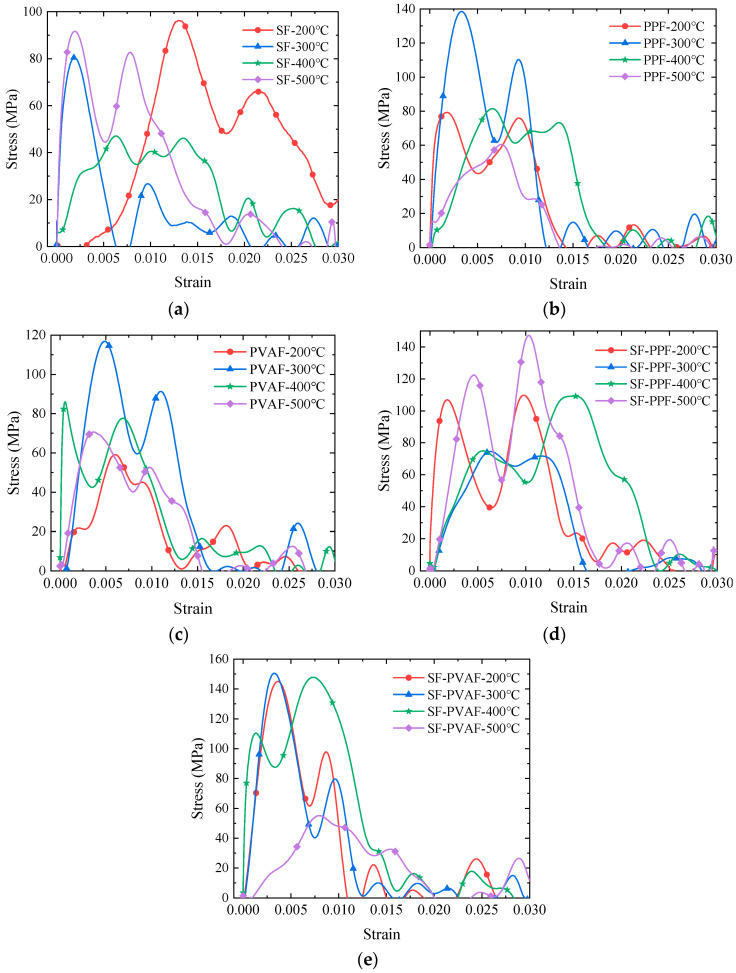
Dynamic stress–strain relationships of different fiber-reinforced high-performance concrete at real-time temperatures of 200–500 °C. (**a**) SFs; (**b**) PPFs; (**c**) PVAFs; (**d**) SF-PPF; (**e**) SF-PVAF.

Figure 5 shows the dynamic stress–strain relationships of HPC reinforced with steel fibers (SFs), polypropylene fibers (PPFs), polyvinyl alcohol fibers (PVAFs), and their hybrid combinations (SF-PPF and SF-PVAF) under real-time temperatures ranging from 200 to 500 °C.

Distinct dynamic compressive behaviors are observed between single-fiber and hybrid-fiber systems under varying thermal conditions. As shown in Figure 5a, the dynamic compressive strength of pure SFs exhibits a V-shaped trend, reaching a minimum at 400 °C. In contrast, the pure PPF and PVAF systems demonstrate an inverted V-shaped trend (Figure 5b,c), with peak dynamic strength observed at 300 °C, highlighting the divergent thermal–shock responses between metallic and organic fibers.

For the hybrid systems (Figure 5d,e), SF-PPF retains a V-shaped strength profile similar to pure SF, while SF-PVAF maintains stable dynamic compressive strength from 200 to 400 °C, followed by a sharp decline at 500 °C. This indicates that the hybridization of steel and PVA fibers enhances dynamic stability within 200–400 °C due to synergistic toughening mechanisms, whereas the complete decomposition of PVA fibers at 500 °C triggers abrupt interfacial failure.

#### 3.2.3. Dynamic Compressive Strength Growth Coefficient

Table 4 contains the dynamic compressive strength growth coefficient data calculated from the static compressive strength and dynamic compressive strength values obtained from the aforementioned experiments. The dynamic impact factor (DIF) can be calculated using the following Formula (6).

*DIF* = *σ_dynamic_*/*σ_static_* (6)

where *σ_dynamic_* is the stress value obtained from the dynamic test (MPa), and *σ_static_* is the stress value obtained from the static test (MPa).

From Table 4, it can be observed that the DIFs for various fiber-reinforced specimens exhibit the following trends: The DIF for SFs continuously decreases from 200 °C to 400 °C (0.88 → 0.43) and then rises to 0.84 at 500 °C. For PPFs, the DIF sharply increases to its peak value (1.75) at 300 °C and then drops to 0.76 at 500 °C. The DIF for PVAFs is lowest at 200 °C (0.62), increases to 1.22 at 300 °C, and then decreases to 0.74 at 500 °C.

When a 1:1 mixture is applied, SF-PPF shows higher coefficients at 200 °C and 400 °C (1.23 and 1.22), with a significant decrease to 0.83 at 300 °C. Meanwhile, SF-PVAF maintains a relatively stable coefficient between 1.65 and 1.68 from 200 °C to 400 °C, followed by a sharp decline to 0.63 at 500 °C. It can be concluded that the performance of hybrid fibers under high-temperature dynamic impact is significantly superior to that of single fibers, with SF-PPF demonstrating notably higher DIF values above 400 °C compared with single fibers. Furthermore, the performance of SF-PVAF remains stable and excellent in the 200 °C to 400 °C range (with little variation in the DIF and a consistently high level), which is primarily attributed to the bridging effect of SFs, the buffering of melting holes, and the carbonized framework provided by PPFs/PVAFs, thus delaying high-temperature damage.

#### 3.2.4. Strain Energy Density (SED)

**Figure 6:**
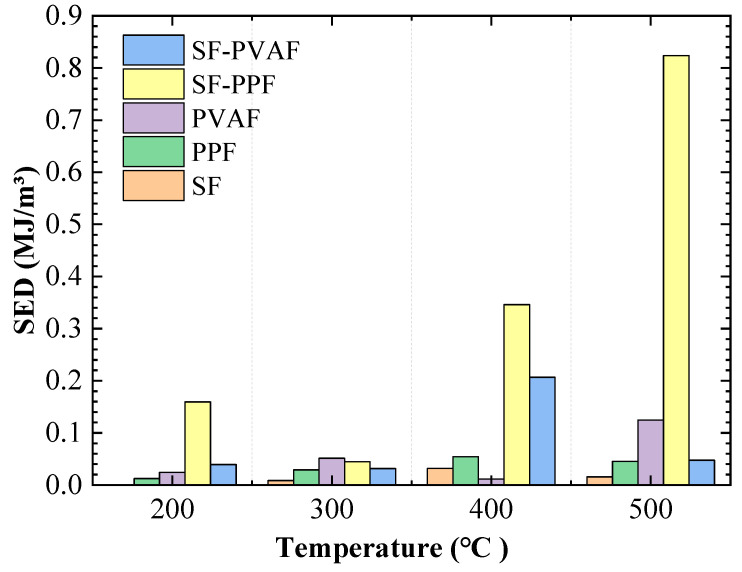
Strain energy density.

To investigate the energy evolution patterns of the specimens under impact at real-time high temperatures with various fiber additions, the SED for each specimen at different real-time temperatures was calculated based on the measured stress–strain data, as illustrated in Figure 6. Integrating the area under the peak of each stress–strain curve shown in Figure 5, the SED per unit volume can be calculated.

Figure 6 shows that the SED value for SFs reaches its maximum at a real-time temperature of 400 °C, while the maximum SED values for PVAFs and SF-PPF occur at a real-time temperature of 500 °C. For PPFs and SF-PVAF, the maximum SED values are observed at a real-time temperature of 400 °C. This indicates a strong temperature-dependent evolution of the SED in hybrid fiber-reinforced concrete.

Compared with the specimens containing only steel fibers, the addition of PP and PVA fibers significantly enhances ductility in the low-temperature range (below 300 °C), resulting in SED values greater than those of SFs. In particular, the SF-PPF hybrid specimen demonstrates superior SED values at high temperatures compared with other fiber combinations, showing rapid increases in the SED. Although there is a temporary decrease in the SED at 300 °C due to interface debonding, the SED remains higher than that of SFs, PPFs, and SF-PVAF.

These trends are primarily influenced by the melting or softening behaviors of the fibers at different temperature ranges; the synergistic toughening mechanism between steel fibers and organic fibers also plays a crucial role.

### 3.3. Microscopic and Macroscopic Failure Modes

**Figure 7:**
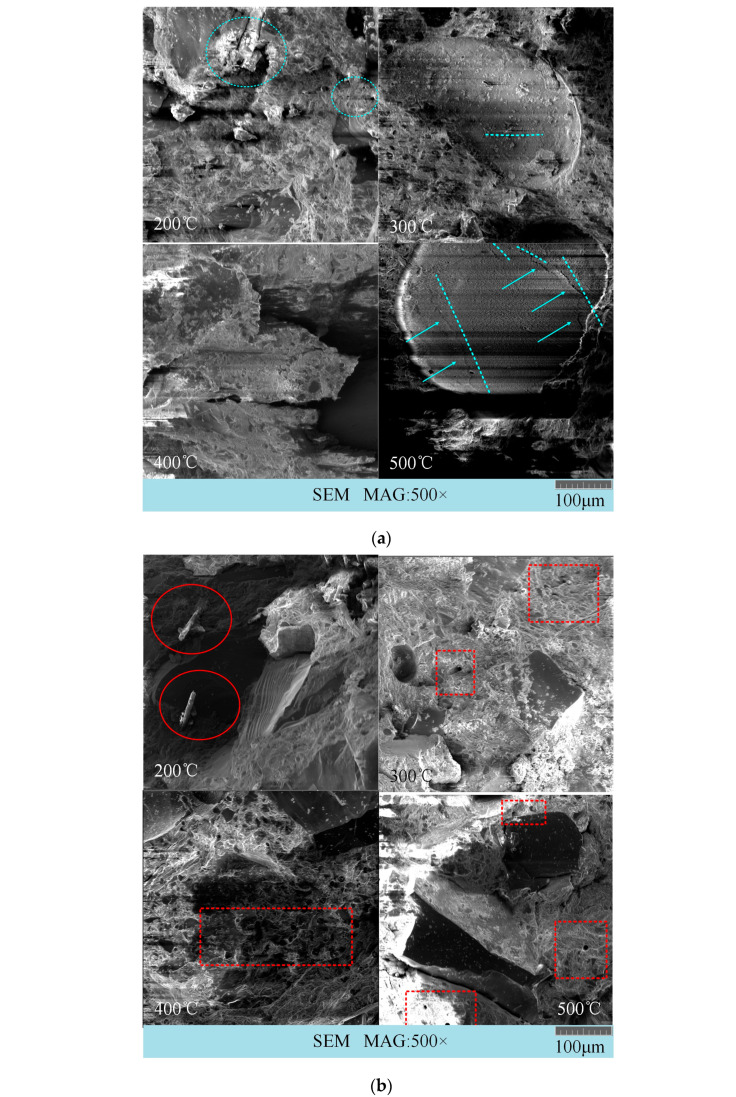

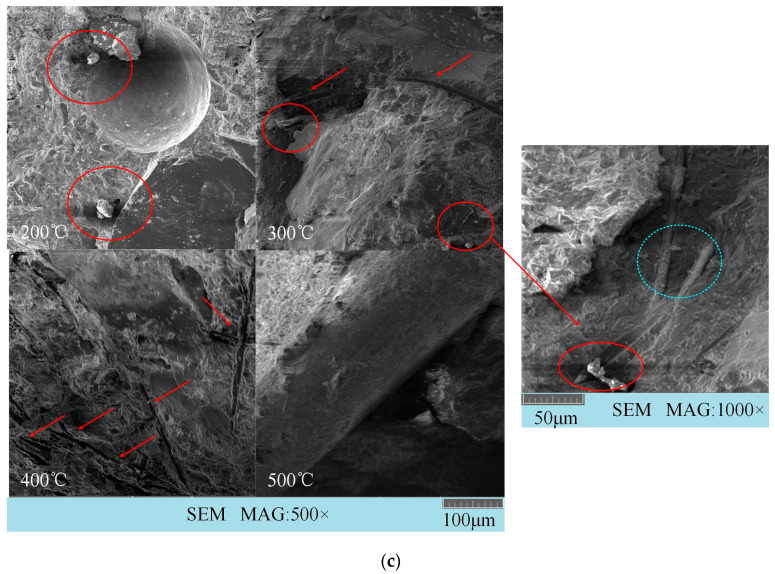
SEM images of specimens after impact at different temperatures. (**a**) SF; (**b**) PVAF; (**c**) SF-PVAF.

For ease of analysis, only three representative fiber combinations—SF (steel fiber), PVAF (polyvinyl alcohol fiber), and SF-PVAF (a hybrid of steel fiber and polyvinyl alcohol fiber)—were selected to examine the SEM micrographs and macroscopic impact failure images after impact at different real-time temperatures. Figure 7 displays the SEM images of the three fiber combinations.

In Figure 7, the blue circles indicate steel fibers, the blue arrows point to micro-cracks, the red circles indicate PVA fibers, the red dashed circles denote holes left after the melting of PVA fibers, and the red arrows point to gaps left after PVA fibers were pulled out or melted.

In Figure 7a, the SEM images of SFs after impacts at 300 °C and 500 °C reveal structures resembling craters, with micro-cracks present inside these craters. This phenomenon indicates brittle fracture caused by stress concentration within the concrete due to the impact, forming irregular depressions. During the dynamic impact process, secondary cracks emerge at the edges of the depressions due to plastic deformation or shear effects, which corresponds to the relatively low SED value of SFs, as mentioned earlier.

In Figure 7b, the SEM image of PVAFs shows that at 200 °C, the PVA fibers have not yet melted, and the bonding between the PVA fibers and the cement interface remains strong. During the impact, the fibers are pulled and break, losing their bridging function. As the temperature rises, the PVA fibers gradually melt, creating a series of holes within the concrete, which can help dissipate part of the impact energy.

Figure 7c illustrates the SEM image of SF-PVAF. At 200 °C, the PVA fibers have not yet melted; at 300 °C, some fibers are still present. The image at 1000× magnification indicates that the bonding between the remaining PVA fibers and the interface becomes very loose at 300 °C. After impact, the PVA fibers are pulled out, losing their bridging function, while the steel fibers remain intact. The SEM image shows that the anchoring of the steel fibers to the interface remains effective, enabling the entire structure to maintain a relatively high strength. At 400 °C, the PVA fibers completely melt, creating numerous channels within the concrete that help absorb the impact energy.

**Figure 8:**
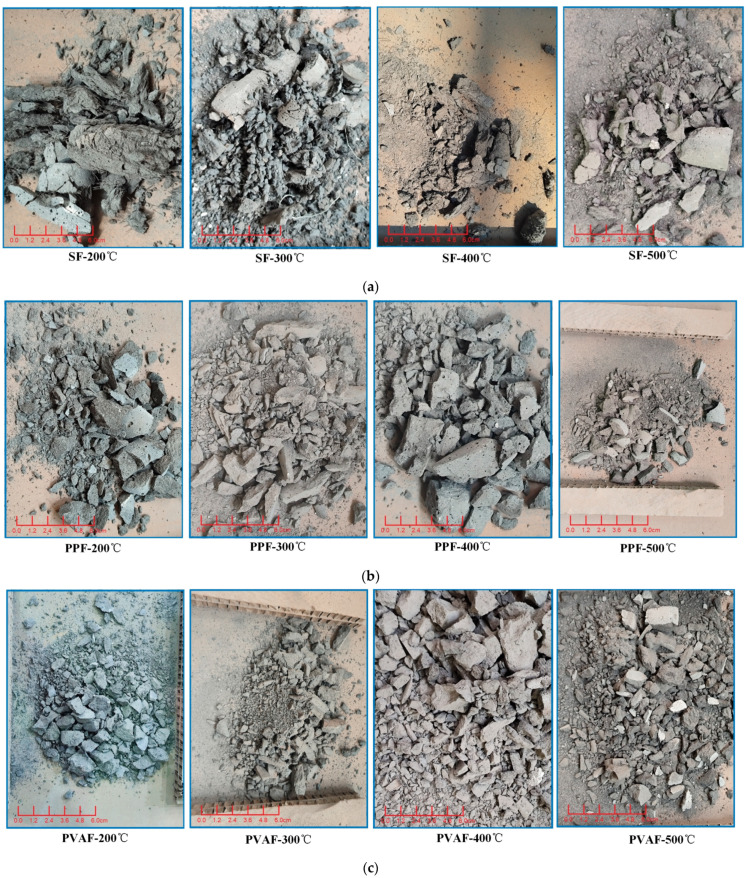

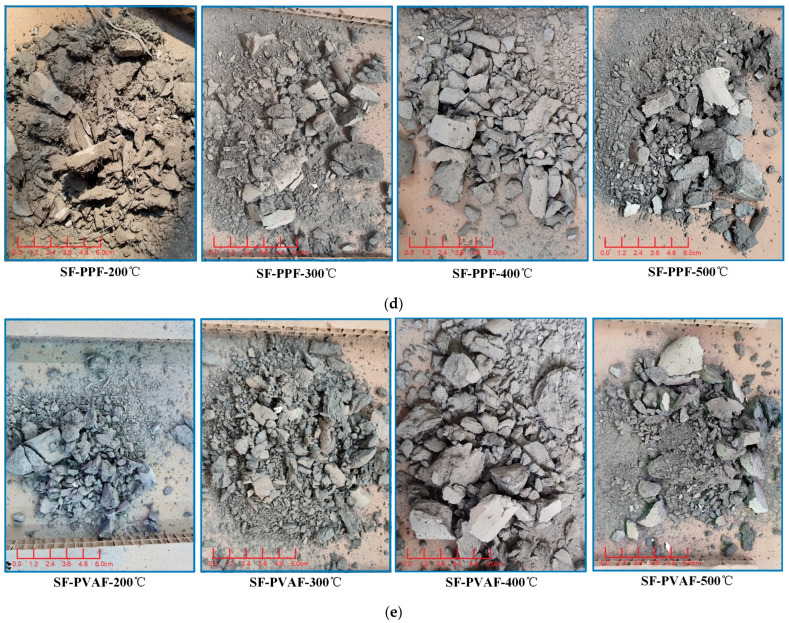
Residual pictures of the specimens after impact at real-time high temperatures. (**a**) SFs; (**b**) PPFs; (**c**) PVAFs; (**d**) SF-PPF; (**e**) SF-PVAF.

Figure 8 shows the residual images collected after high-temperature impacts on the specimens.

The figure shows that there are similarities in the debris morphology of the different fiber composite specimens after impacts at 200 °C and 500 °C. At 200 °C, the fracture morphology of each specimen is primarily characterized by large particles with sharp edges, and the color of the specimens is gray. In contrast, at 500 °C, a higher proportion of smaller particle fragments is observed, and the surfaces of the specimens appear yellow, indicating that complex chemical reactions occurred at high temperatures, including fiber carbonization and the decomposition of C-S-H gel, leading to a change in the color of the fragments.

As the temperature increases, the specimens containing steel fibers and those containing PP and PVA fibers gradually exhibit different failure modes. Comparing the debris images of each specimen in Figure 8, which were taken at temperatures ranging from 200 °C to 500 °C, it can be seen that for the SF specimens, as the temperature rises, the broken morphology after high-temperature impacts becomes increasingly discrete. At 200 °C, the residual material is relatively intact, and a clear steel fiber skeleton can be seen. At 300 °C, the residues become more fragmented, but the skeleton is still visible. At 400 °C, some skeleton remains, and at 500 °C, although large fragments are present, the overall structure no longer shows the steel fiber skeleton (Figure 8a).

Figure 8d,e show that the SF-PPF and SF-PVAF specimens containing 1% steel fibers have residues at 200 °C that are similar to those of the SF specimens, being relatively intact, with steel fibers visibly attached to the residual debris, indicating the presence of a steel fiber skeleton. However, as the temperature increases, it becomes difficult to identify the steel fiber skeleton in the images. The PP specimens (Figure 8b) and the PVA specimens (Figure 8c), which do not contain steel fibers, show no fiber skeletons in the images of the debris after impacts at temperatures between 200 °C and 500 °C; additionally, their failure modes are more fragmented compared with those containing steel fibers.

Examination of the residual fragments of each specimen at 500 °C in Figure 8 shows that after mixing steel fibers with PP and PVA fibers, most of the organic fibers melt at high temperatures. Under the impact load, only about 1% of the residual steel fibers remain to bear the load. Therefore, based on the fracture morphology, it can be seen that even under the same impact load at 500 °C, only the pure steel fiber specimens have larger residual particles, while the debris from PPFs and PVAFs primarily consists of smaller particles. In the 1:1 hybrid case, the fragmentation of SF-PPF and SF-PVAF lies between that of the specimens with 2% organic fibers and those with 2% steel fibers. This suggests that the pure SF specimens mainly exhibit brittle failure, while the specimens incorporating organic fibers produce finer debris due to the energy absorption effect of the pores created after melting.

## 4. Discussion

### 4.1. Synergistic Effects of Hybrid Fibers at Room Temperature

As shown in Section 3.1, Figure 4, and Table 3, the addition of fibers at room temperature significantly enhances the uniaxial compressive strength, three-point flexural strength, and tensile strength of concrete. However, the enhancement effects of different fiber types on various strength indices exhibit notable differences, revealing the diversity of the toughening mechanisms of fibers.

#### 4.1.1. Uniaxial Compressive Strength

As shown in Section 3.1, Table 3, the plain concrete (PC) specimens exhibited a limited uniaxial compressive strength of 44.87 MPa. This is mainly attributed to the use of aggregate with a narrow particle size distribution (40–70 mesh quartz sand), which results in a relatively uniform particle distribution, reducing particle packing density and weakening the skeleton effect. Meanwhile, the amount of cementitious materials was relatively low (1060 kg/m^3^ total, including 900 kg/m^3^ cement and 160 kg/m^3^ silica fume), insufficient to form a solid matrix structure. In addition, although curing in hot water at 70 °C accelerated the pozzolanic reaction of silica fume, it also generated localized thermal and moisture gradients, leading to internal defects. These factors together weakened the load-bearing capacity of the PC.

In contrast, the incorporation of fibers significantly enhanced the mechanical properties (Table 3). The addition of steel fibers increased the uniaxial compressive strength of the plain concrete by 143.44%, significantly higher than that of the other fiber types. This is mainly due to the high elastic modulus of steel fibers and their mechanical interlocking effect with the cement matrix. The bridging effect of steel fibers effectively suppressed lateral deformation of the specimens during uniaxial compression. Although the compressive strengths of SF-PPF and SF-PVAF (89.39 MPa and 87.85 MPa, respectively) were lower than that of the pure steel fiber (SF) mixture, they were still significantly higher than that of the pure polypropylene fibers (PPFs) (79.31 MPa). It is noteworthy that the compressive strength of the hybrid steel fiber and PVA fiber mixture (SF-PVAF) was lower than that of the mixtures using SFs or PVAFs alone, which may be due to excessive squeezing of the steel fibers by the PVA fibers, thereby weakening the bridging effectiveness of the steel fibers.

#### 4.1.2. Flexural Strength

Table 3 shows that the flexural strength of the SF-PVAF specimen reaches 24.86 MPa (an increase of 79.62%), exceeding that of pure SFs (21.9 MPa). This indicates that the mixed addition of steel and PVA fibers, while somewhat weakening the uniaxial compressive strength, enhances the flexural strength of the specimens. The addition of other fibers also contributes to an increase in the flexural strength of plain concrete to some extent. The flexural strength of the PPF specimen only increased by 6.79%, which is attributed to its low surface energy, resulting in weak interfacial bonding. The hydrophobic nature of PPF restricts its chemical bonding with the hydration products of cement, making its bridging action primarily dependent on mechanical anchoring.

#### 4.1.3. Tensile Strength

Table 3 indicates that the tensile strength of the SF specimen (11.63 MPa) is increased by 240.06%, corroborating the effective bridging capability of steel fibers under tensile loading. However, the tensile strength improvement rates for SF-PPF and SF-PVAF (26.32% and 54.68%, respectively) are significantly lower than those of pure SF (240.06%) or pure PPF (44.74%) and pure PVAF (73.68%). This may be due to the premature failure of the organic fibers during the Brazilian splitting test.

### 4.2. Temperature-Dependent Dynamic Behavior

#### 4.2.1. Dynamic Compressive Strength Transition

**Figure 9:**
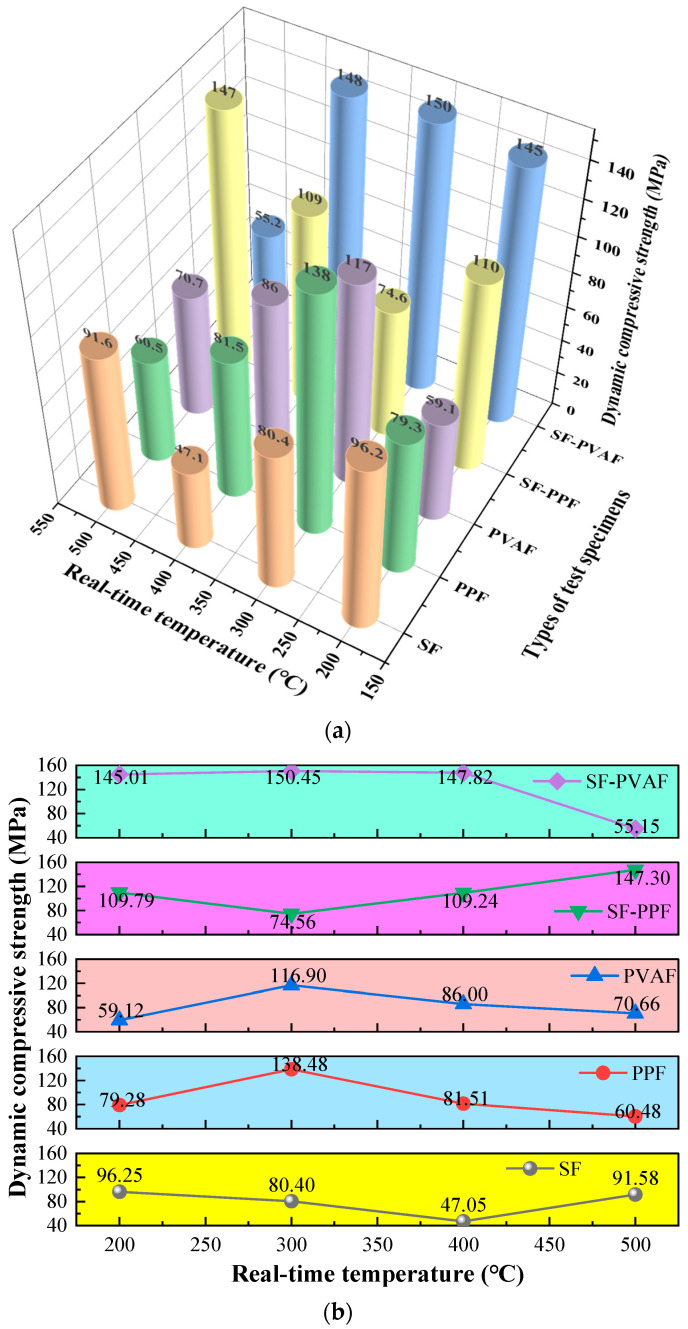
Dynamic compressive strengths of different fiber-reinforced high-performance concrete at real-time temperatures of 200–500 °C. (**a**) Overview of the dynamic compressive strength of each specimen; (**b**) Comparison of the dynamic compressive strength of specimens made from different materials at various temperatures.

To facilitate visual analysis and discussion, the dynamic compressive strengths of each specimen summarized in Figure 5 are presented in Figure 9.

The V-shaped trends in the dynamic compressive strengths of SFs and SF-PPF, as well as the inverted V-shaped trend for PPFs/PVAFs, are clearly observed in Figure 9b. The reason for the V-shaped trends in the dynamic compressive strengths of SFs and SF-PPF with temperature variations lies in the fact that as the temperature increases, micro-cracks induced by the initial thermal expansion continuously propagate, while the steel fibers exhibit softening phenomena. The dynamic compressive strength of the specimens is lowest when the temperature reaches 300 °C for SF-PPF and 400 °C for SF. However, as the temperature continues to rise, partial oxidation and hardening occur within the specimens, improving their dynamic compressive strength. The differing thresholds for SF-PPF and SF are primarily due to the softening and melting of PPF fibers around 200 °C, resulting in an earlier threshold at 300 °C. However, the formation of a series of micro-porous structures within the specimens absorbs impact energy, enhancing the dynamic compressive strength after 300 °C. In contrast, the inverted V-shaped curves for PPFs/PVAFs (Figure 9b) reflect the thermal activation of organic fibers. At 300 °C, the melting of PPF/PVA forms micro-porous structures that absorb impact energy through viscous flow. Above 400 °C, carbonized residues form a brittle framework, thereby reducing the overall mechanical performance. The dynamic compressive strength of SF-PVAF remains relatively stable between 200 °C and 400 °C, indicating that within this temperature range, the bridging of macro-cracks by SFs and the filling of micro-pores by the carbonized products of PVAFs delays thermal spalling, achieving synergy between the two fibers. The decrease in performance at 500 °C is also attributed to the formation of a brittle framework from carbonized residues, which reduces the overall mechanical performance.

#### 4.2.2. Discussion of the Law of the Dynamic Compressive Strength Growth Coefficient

**Figure 10:**
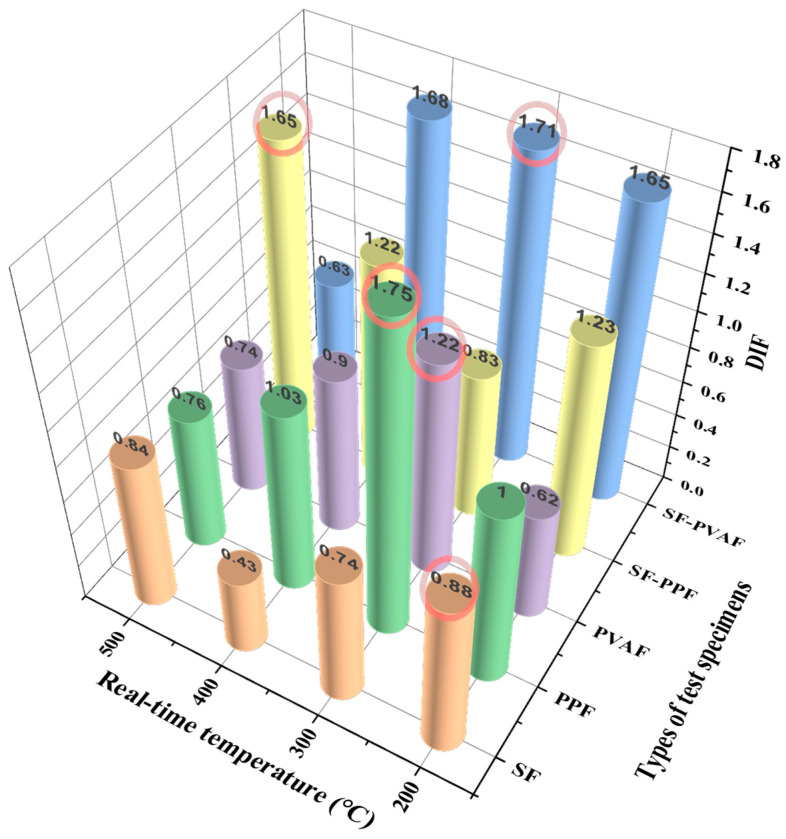
DIF values of different fiber-reinforced high-performance concrete at real-time temperatures of 200–500 °C.

For a clearer analysis, the DIF values of each specimen in Table 4 are summarized in Figure 10.

As the real-time temperature varies, the DIF values of the specimens with different fiber compositions exhibit different change mechanisms. The patterns of variation in the DIF values for the single fibers are consistent with the aforementioned variations in dynamic compressive strength, mainly attributed to the coordination of micro-crack propagation induced by thermal expansion, fiber bridging, energy absorption from channels formed after melting, and hardening due to high-temperature carbonization.

For the hybrid fibers, the DIF values of SF-PVAF remain very close between 200 °C and 400 °C, demonstrating excellent stability. This aligns with the conclusion in Section 4.2.1 that the bridging effect of steel fibers on macro-cracks synergizes with the carbonized products and channels formed by the melting of PVAFs. The sudden decrease in the DIF value at 500 °C is attributed to the complete decomposition of PVAFs, leaving behind a weakened steel fiber framework. In contrast, SF-PPF maintains a higher DIF value due to the formation of a stable porous structure from the lower melting point of PPFs, which alleviates stress concentration.

### 4.3. Energy Dissipation Mechanisms

Analyzing the changes in the SED with real-time temperature (as shown in Section 3.2.4) indicates that, as the temperature increases, the melting of organic fiber materials generates channels within the concrete that can dissipate more impact energy. Simultaneously, the softening of the material itself due to increasing temperature can also absorb some energy through plastic deformation. However, as the temperature rises, the interface between the fibers and the matrix deteriorates, reducing the material’s crack resistance. The residual steel fibers still provide some bridging effect at 400 °C and 500 °C. Therefore, the hybridization of SF with PPF or PVAF under high-temperature impacts exhibits superior mechanical performance compared with single fibers. However, because of the differences in the properties of organic materials, the optimal temperature for fiber combinations varies.

Figure 6 shows that the SED value of SF-PPF reaches a minimum at 300 °C, which shows a significant difference from the development trends of the SED values of SFs and PPFs. This is because at 200 °C, the PP fibers have just begun to melt, and by 300 °C, the melting process is essentially complete. The remaining material continues to oxidize within the pores, weakening the anchoring strength of the steel fibers. At 300 °C, the steel fibers also begin to soften, further reducing their anchoring strength. The combined effects of these two factors result in a low SED value for the fiber composite at 300 °C. As the temperature increases, the reactions occurring within the pores created by the PP fibers tend to stabilize, enhancing the energy absorption capability and leading to an increase in the SED value.

### 4.4. Correlation Between Macroscopic and Microscopic Failure Modes

A comparative analysis of Figure 7 and Figure 8 shows that the surface of the steel fibers exhibits crater-like depressions after impacts at 300 °C and 500 °C, accompanied by micro-cracks. This morphology indicates brittle spalling occurring in regions of stress concentration during dynamic impact, forming localized depressions. In Figure 8, the fragments of the pure SF specimen at 500 °C are primarily large and plate-like, with sharp edges, confirming the dominance of brittle failure modes.

Analyzing the PVAFs, Figure 7b shows that at 200 °C, PVA fibers maintain a strong bond with the matrix, providing ductility through fiber pull-out. However, at 400 °C and above, the complete decomposition of PVAFs results in carbonized residual voids, which, despite the loss of bridging function, can still buffer and absorb some energy through the pores. This indicates that energy dissipation occurs through multi-scale mechanisms (void buffering + fiber pull-out) rather than concentrated brittle fractures, which aligns with the observation in Figure 8c that the fragments of PVAF are primarily small particles.

For SF-PVAF, combining the previous SED trends indicated in Figure 7c, we find that the carbonized skeleton of PVAF synergizes with SF bridging between 200 °C and 400 °C, maintaining a stable SED of 1.65–1.68. Below 300 °C, the combination of the two fibers improves the brittleness of pure SFs. This is reflected in Figure 8c, where the fragments from SF-PVAF after impact at high temperatures fall between those of SFs and PVAFs.

### 4.5. Engineering Implications

For fire-resistant structures experiencing transient thermal shocks (200–400 °C), SF-PVAF hybrid systems offer optimal dynamic performance. In sustained high-temperature environments (>400 °C), SF-PPF combinations provide better post-fire impact resistance. The critical 300 °C threshold identified for organic fiber phase changes suggests the need for temperature-adaptive fiber selection in design codes. For structures exposed to temperatures between 200 and 400 °C, the SF-PVAF hybrid can provide optimal dynamic performance. For structures exposed to temperatures above 400 °C and below 500 °C, selecting the SF-PPF combination can offer better dynamic performance.

### 4.6. Limitations and Future Directions

This study systematically reveals the dynamic response mechanisms of hybrid fiber-reinforced HPC at real-time high temperatures ranging from 200 to 500 °C. However, it has several limitations:

(1) The temperature range in this study is limited to 200–500 °C, which restricts its applicability to certain industrial scenarios and lacks guidance for industrial environments at higher temperatures. Additionally, the formulation of the specimens studied (40–70 mesh quartz sand gradation) and the fiber reinforcement scheme (a uniform 1:1 volume ratio mix) were relatively simplistic, making it difficult to determine the materials’ behaviors in complex engineering environments. Future research will expand the temperature range to 800 °C to encompass more high-temperature industrial scenarios and include additional fiber mix ratios to explore the threshold of fiber synergistic effects.
(2) The singular gradation (40–70 mesh quartz sand) led to a relatively low strength of the plain concrete specimens (44.87 MPa), which limited the benchmark evaluation of the fiber reinforcement effects. Future studies will optimize aggregate gradation by utilizing continuously graded quartz sand for specimen casting and improve the curing process by introducing steam curing techniques to enhance the density of the matrix.
(3) The SHPB excitation pressure (0.2 MPa) resulted in a uniform strain rate. Subsequent research will investigate different strain rates corresponding to various scenarios by setting different SHPB excitation pressures, thereby revealing the coupling mechanisms between strain rate effects and real-time temperature.

## 5. Conclusions

This study systematically investigated the effects of steel fibers, PP fibers, PVA fibers, and their 1:1 hybrid systems on the static and dynamic mechanical properties, energy dissipation mechanisms, and failure modes of HPC at room temperature and real-time high temperatures (200–500 °C). The results revealed the regulatory roles of the fiber type and hybrid ratio on the multi-scale mechanical behaviors of concrete and allowed for the proposal of fiber adaptation design principles for environments between 200 and 500 °C. The main conclusions are as follows:

1. Enhancement of Compressive/Tensile Strength: Steel fibers (SFs) significantly enhance the compressive and tensile strengths of concrete, increasing them by 143.44% and 240.06%, respectively. The high elastic modulus and bridging effect of SFs dominate the effective stress transfer. Meanwhile, the SF-PVAF combination showed the best improvement in flexural strength, reaching 79.62%, due to the synergistic effect of macro-crack bridging by steel fibers and micro-crack branching suppression by PVA fibers.
2. Evolution Pattern of Dynamic Compressive Strength: Under real-time temperature conditions, the dynamic compressive strength of SFs and SF-PPF exhibits a “V-shaped” evolution pattern, while those of PPFs and PVAFs show an “inverted V-shaped” trend, influenced by matrix thermal expansion damage–matrix oxidation hardening and fiber melting–carbonization skeleton effects, respectively. The dynamic compressive strength of SF-PVAF remains stable (DIF ≈ 1.65) at 200–400 °C, with the bridging function of steel fibers and the melting and carbonization behavior of PVA working together to enhance toughness. At 500 °C, SF-PPF maintained a high DIF value (1.65), attributed to the stable porous structure formed by melting holes in PP. The peak SED value for steel fibers occurred at 400 °C, while the highest SED value for SF-PPF at 500 °C resulted from the energy absorption effect of melting holes, reflecting the diversity of energy dissipation paths in hybrid fibers.
3. Dynamic Fragmentation Mechanism: The dynamic fracture mechanisms at real-time temperatures revealed that the SF system exhibits a micro “meteorite crater” morphology, indicating that stress concentration leads to brittle spalling, which corresponds to large debris on a macroscopic scale. The low SED value indicates that energy is primarily dissipated through localized fragmentation. In the organic fiber system, melting holes of PVA (200–400 °C) and carbonization channels (500 °C) dissipate energy through crack deflection and multiple branching, resulting in smaller debris particles. The debris size of the SF-PVAF hybrid fiber system lies between those of pure SFs and PVAFs, improving the brittle failure characteristics of pure SFs.
4. Recommendation for Optimal Dynamic Performance: Structures exposed to environmental temperatures of 200–400 °C can achieve optimal dynamic performance with the SF-PVAF hybrid mix during transient thermal shocks. When the environmental temperature reaches 400–500 °C, the SF-PPF combination is recommended to enhance the impact resistance and high-temperature performance of the material.

## Figures and Tables

**Table 1:** Test base formula.

Material	Specification	Mass per Unit Volume (kg/m^3^)
Cement	P∙Ⅱ52.5 Portland cement	900.00
Quartz sand	High-purity, 40–70 mesh	940.00
Silica fume	Premium grade	160.00
Additive	Polycarboxylate superplasticizer	17.00
Water	Deionized water	190.00
Fibers	SF/PP/PVA fibers (12 mm length)	Volume fraction: 2%

**Table 2:** Specimen groupings.

Group Designation	Types of Fiber Reinforcement	Fiber Volume Fraction (vol.%)
PC	No fiber, plain concrete	0
SF	Steel fiber	2
PPF	PP fiber	2
PVAF	PVA fiber	2
SF-PPF	Steel + PP fiber	1 + 1
SF-PVAF	Steel + PVA fiber	1 + 1

**Table 3:** Static strength index.

Strength/Type of Specimen	PC	SF	PPF	PVAF	SF-PPF	SF-PVAF
Single-axis compressive strength (MPa)	44.87	109.23	79.31	95.99	89.39	87.85
Triaxial flexural strength (MPa)	13.84	21.9	14.78	17.39	19.66	24.86
Tensile strength (MPa)	3.42	11.63	4.95	5.94	4.32	5.29
Compression strength improvement rate (%)	-	143.44	76.76	113.93	99.22	95.79
Triaxial flexural strength improvement rate (%)	-	58.24	6.79	25.65	42.05	79.62
Tensile strength improvement rate (%)	-	240.06	44.74	73.68	26.32	54.68

**Table 4:** Dynamic compressive strength growth coefficient.

Temperature (°C)/Type of Specimen	SFs	PPFs	PVAFs	SF-PPF	SF-PVAF
200	0.88	1.00	0.62	1.23	1.65
300	0.74	1.75	1.22	0.83	1.71
400	0.43	1.03	0.90	1.22	1.68
500	0.84	0.76	0.74	1.65	0.63

## Data Availability

The original contributions presented in this study are included in the article. Further inquiries can be directed to the corresponding author.

## References

[1] Sohail M.G., Kahraman R., Al Nuaimi N., Gencturk B., Alnahhal W. (2021). Durability characteristics of high and ultra-high performance concretes. J. Build. Eng..

[2] Ozyildirim H.C., Sharifi M. (2022). High-Performance Fiber Reinforced Concretes in Virginia. Transp. Res. Rec..

[3] Doostkami H., de Jesús Estacio Cumberbatch J., Formagini S., Serna P., Roig-Flores M. (2023). Self-healing capability of conventional, high-performance, and Ultra High-Performance Concrete with commercial bacteria characterized by means of water and chloride penetration. Constr. Build. Mater..

[4] Dong S., Gu J., Ouyang X., Jang S.-H., Han B. (2025). Enhancing mechanical properties, durability and multifunctionality of concrete structures via using ultra-high performance concrete layer: A review. Compos. Part B Eng..

[5] Wen Y., Wang Z., Yuan X., Yang X. (2025). Optimization of Mechanical Properties and Durability of Steel Fiber-Reinforced Concrete by Nano CaCO3 and Nano TiC to Improve Material Sustainability. Sustainability.

[6] He J., Wang H., Yu Q. (2025). Flexural fracture behavior of ultra-high performance concrete after high-temperature exposure. Constr. Build. Mater..

[7] Agrawal S., Yulianti E., Amran M., Hung C.-C. (2025). Behavior of unconfined steel-fiber reinforced UHPC post high-temperature exposure. Case Stud. Constr. Mater..

[8] Tai Y.-S., Lee M.-H. (2025). Tensile behavior and damage mechanisms of ultra-high-performance concrete with blended steel fibers under elevated temperatures. J. Build. Eng..

[9] Felicetti R., Yarmohammadian R., Pont S.D., Tengattini A. (2024). Fast vapour migration next to a depressurizing interface: A possible driving mechanism of explosive spalling revealed by neutron imaging. Cem. Concr. Res..

[10] Zhang T., Zhu H., Zhou L., Yan Z. (2021). Multi-level micromechanical analysis of elastic properties of ultra-high performance concrete at high temperatures: Effects of imperfect interface and inclusion size. Compos. Struct..

[11] Li Y., Tan K.H., Yang E.-H. (2018). Influence of aggregate size and inclusion of polypropylene and steel fibers on the hot permeability of ultra-high performance concrete (UHPC) at elevated temperature. Constr. Build. Mater..

[12] Zhao X., Lu J.-X., Tian W., Cyr M., Tagnit-Hamou A., Poon C.S. (2025). Self-healing performance of thermally damaged ultra-high performance concrete: Rehydration and recovery mechanism. Cem. Concr. Res..

[13] Wang F., Xiong T., Liu J., Zeng J., Luo C. (2025). Experimental study on temperature characteristics and multi-field analysis for concrete spalling of tunnel linings exposed to high temperatures. Struct. Concr..

[14] Shu R., Cheng J., Xu G., Lai Y., Huang L. (2024). Dynamic tensile properties of thermally treated concrete specimens subjected to varied heating rates: An investigation using the digital image correlation method. Mech. Time-Depend. Mater..

[15] Zhang H., Zhang W., Chen Y., Chen R., Liu Y., Zhang Y. (2024). Study on the dynamic impact mechanical properties of high-temperature resistant ultra-high performance concrete (HTRUHPC) after high temperatures. J. Build. Eng..

[16] Yang J., Yan K., Doh J.-H., Zhang X. (2023). Experimental study on shear performance of ultra-high-performance concrete beams at elevated temperatures. Eng. Struct..

[17] Zhang Z., Abdalla J.A., Yu J., Chen Y., Hawileh R.A., Mahmoudi F. (2025). Use of polypropylene fibers to mitigate spalling in high strength PE-ECC under elevated temperature. Case Stud. Constr. Mater..

[18] Khan M., Lao J., Ahmad M.R., Dai J.-G. (2024). Influence of high temperatures on the mechanical and microstructural properties of hybrid steel-basalt fibers based ultra-high-performance concrete (UHPC). Constr. Build. Mater..

[19] Shahidzadeh Arabani A., Dashti Naserabadi H., Aminyavari S. (2025). Experimental investigation of energy absorption in fiber-reinforced ultra high-performance concrete after exposure to elevated temperatures. Case Stud. Constr. Mater..

[20] Xiao L., Yan H., Zhu Z., Xu C. (2024). Mechanical Performance of Steel-PVA Hybrid Fiber Concrete After Elevated Temperature Exposure. Arab. J. Sci. Eng..

[21] Hung C.-C., Yulianti E., Agrawal S. (2024). Microstructures, durability, and mechanical behavior of hybrid steel and PP fiber reinforced UHPC at elevated temperatures. Constr. Build. Mater..

[22] Abed F., Khalaf S., Alhoubi Y., Moustafa M.A., Al Jamal M. (2024). Effect of fiber types on fire-induced spalling and thermal performance of UHPC circular columns. Dev. Built Environ..

[23] Lin J., Zhang Y., Huang S., Du H., Jiang K. (2024). Influence of synthetic fibers on the performance of ultra-high performance concrete (UHPC) at elevated temperatures. J. Build. Eng..

[24] Lin J., Zhang Y., Guo Z., Du H. (2024). Impact of synthetic fibers on spalling and intrinsic pore structure of ultra-high performance concrete (UHPC) under elevated temperatures. Constr. Build. Mater..

[25] Dziomdziora P., Smarzewski P. (2025). Effect of Hybrid Fiber Compositions on Mechanical Properties and Durability of Ultra-High-Performance Concrete: A Comprehensive Review. Materials.

[26] Wang T., Yu M., Tian J., Sun Z., Yu C., Ye J. (2025). Residual properties of ultra-high performance concrete containing steel-polypropylene hybrid fiber exposed to elevated temperature at early age. J. Build. Eng..

[27] Shen X., Li X., Liu L., Chen X., Du J. (2024). Research on Mechanical Properties of Steel-Polypropylene Fiber-Reinforced Concrete after High-Temperature Treatments. Appl. Sci..

[28] Xu Z., He T., Liu Y., Chen X., Liu L. (2022). Study on Dynamic Splitting Properties of S-PP Hybrid Fiber Concrete after High Temperatures. Appl. Sci..

[29] Qiu H., Lai H., Liao F., Chen Y. (2024). Experimental study on dynamic fracture of UHPC-NC specimens after high temperature burning treatment. Theor. Appl. Fract. Mech..

[30] Zhuang J., Ni P., Chen J., Wu M. (2023). Study on the impact resistance of steel fiber-reinforced self-compacting concrete after high temperature. Structures.

[31] Chen M., Wang Y., Zhang T., Zhang M. (2023). Microstructural evolution and dynamic compressive properties of engineered cementitious composites at elevated temperatures. J. Build. Eng..

[32] Ping Q., Wu M., Yuan P., Su H., Zhang H. (2020). Dynamic Splitting Experimental Study on Sandstone at Actual High Temperatures under Different Loading Rates. Shock Vib..

[33] Zhang L., Li B., Wu P., Guo S., Zheng Y., Li M., Zhu F. (2024). Experimental Study on the Dynamic Mechanical Properties and Crashing Behaviors of Limestone Under High Temperatures in Real-Time. Appl. Sci..

[34] Li Y., Zhai Y., Xie Y., Meng F. (2023). Research on the Impact Mechanical Properties of Real-Time High-Temperature Granite and a Coupled Thermal–Mechanical Constitutive Model. Materials.

[35] Li M., Zhu F., Mao Y., Fan F., Wu B., Deng J. (2025). Dynamic Mechanical Characteristics and Fracture Size Effect of Coal Sandstone Under High-Temperature and High-Strain Rate Coupling Action. Fractal Fract..

[36] Wu P., Zhang L., Li B., Zheng Y., Li M., Zhu F. (2024). Mechanical properties and microscopic damage characteristics of coal series limestone under coupling effects of high temperature and impact. Sci. Rep..

[37] Li R., Liu L., An H., Wang Y. (2022). Study on Dynamic Constitutive Model of Polypropylene Concrete under Real-Time High-Temperature Conditions. Appl. Sci..

[38] Sucharda O., Gandel R., Cmiel P., Jerabek J., Bilek V. (2024). Utilization of High-Performance Concrete Mixtures for Advanced Manufacturing Technologies. Buildings.

[39] Yang Y., Li Q., Qiao L. (2023). Review of SHPB Dynamic Load Impact Test Characteristics and Energy Analysis Methods. Processes.

